# Machine learning-based models for predicting clinical outcomes after surgery in unilateral primary aldosteronism

**DOI:** 10.1038/s41598-022-09706-8

**Published:** 2022-04-06

**Authors:** Hiroki Kaneko, Hironobu Umakoshi, Masatoshi Ogata, Norio Wada, Takamasa Ichijo, Shohei Sakamoto, Tetsuhiro Watanabe, Yuki Ishihara, Tetsuya Tagami, Norifusa Iwahashi, Tazuru Fukumoto, Eriko Terada, Shunsuke Katsuhara, Maki Yokomoto-Umakoshi, Yayoi Matsuda, Ryuichi Sakamoto, Yoshihiro Ogawa

**Affiliations:** 1 Department of Medicine and Bioregulatory Science, Graduate School of Medical Sciences, Kyushu University, 3-1-1 Maidashi Higashi-ku, Fukuoka, 812-8582 Japan; 2 Department of Diabetes and Endocrinology, Sapporo City General Hospital, Sapporo, Japan; 3 Department of Diabetes and Endocrinology, Saiseikai Yokohamashi Tobu Hospital, Yokohama, Japan; 4 Department of Metabolism and Endocrinology, National Hospital Organization Kyushu Medical Center, Fukuoka, Japan; 5 Department of Endocrinology and Metabolism, National Hospital Organization Kyoto Medical Center, Kyoto, Japan

**Keywords:** Adrenal gland diseases, Adrenal glands

## Abstract

Unilateral subtype of primary aldosteronism (PA) is a common surgically curable form of endocrine hypertension. However, more than half of the patients with PA who undergo unilateral adrenalectomy suffer from persistent hypertension, which may discourage those with PA from undergoing adrenalectomy even when appropriate. The aim of this retrospective cross-sectional study was to develop machine learning-based models for predicting postoperative hypertensive remission using preoperative predictors that are readily available in routine clinical practice. A total of 107 patients with PA who achieved complete biochemical success after adrenalectomy were included and randomly assigned to the training and test datasets. Predictive models of complete clinical success were developed using supervised machine learning algorithms. Of 107 patients, 40 achieved complete clinical success after adrenalectomy in both datasets. Six clinical features associated with complete clinical success (duration of hypertension, defined daily dose (DDD) of antihypertensive medication, plasma aldosterone concentration (PAC), sex, body mass index (BMI), and age) were selected based on predictive performance in the machine learning-based model. The predictive accuracy and area under the curve (AUC) for the developed model in the test dataset were 77.3% and 0.884 (95% confidence interval: 0.737–1.000), respectively. In an independent external cohort, the performance of the predictive model was found to be comparable with an accuracy of 80.4% and AUC of 0.867 (95% confidence interval: 0.763–0.971). The duration of hypertension, DDD of antihypertensive medication, PAC, and BMI were non-linearly related to the prediction of complete clinical success. The developed predictive model may be useful in assessing the benefit of unilateral adrenalectomy and in selecting surgical treatment and antihypertensive medication for patients with PA in clinical practice.

## Introduction

Primary aldosteronism (PA) is a common cause of secondary hypertension, accounting for 3% to 13% of primary care hypertensive patients^1,2^. Patients with PA have higher cardiovascular morbidity and mortality than age- and sex-matched patients with essential hypertension^3,4^. PA is typically classified into unilateral and bilateral subtypes. Most patients with unilateral subtype of PA can be biochemically cured after unilateral adrenalectomy, with complete resolution of hyperaldosteronism. However, according to the Primary Aldosteronism Surgical Outcome (PASO) study, only 37% (16–63%) of patients with unilateral subtype of PA achieved complete clinical success after unilateral adrenalectomy, defined as normalization of blood pressure without antihypertensive medication^5^. Therefore, it is important for both clinicians and patients considering unilateral adrenalectomy to be aware of the predictors of complete clinical success early in the diagnostic process of PA.

Several previous studies have reported preoperative predictors of clinical outcomes such as age, sex, body mass index (BMI), duration of hypertension, use of preoperative antihypertensive drugs, medical history of diabetes, plasma aldosterone concentration (PAC), target organ damage and adrenal nodule size on imaging^6–12^. Accordingly, the likelihood of persistent hypertension after unilateral adrenalectomy is complicated, and the predictors are not consistent across studies, which may impede clinical application. This may be because it is not well understood how predictors affect clinical outcomes.

Machine learning provides techniques that can automatically build computational models of complex relationships between observable variables and relevant objective variables by processing the available data, thus maximizing performance criteria^13^. Traditionally, generalized linear models have been used as statistical methods to make predictions. However, in clinical practice, the relationship between observable and objective variables is often non-linear. Machine learning models based on trees are the most popular non-linear models in use today^14^. Indeed, we and others have recently developed machine learning models for the diagnostic prediction of PA^15–17^. If the relationship between preoperative predictors and clinical outcomes is non-linear, the machine learning approach may be more appropriate for predicting postoperative outcomes in PA.

This study aimed to develop optimal machine learning-based models for predicting postoperative hypertensive remission in patients with PA using preoperative predictors readily available in routine clinical practice and to evaluate the relationship between predictors and clinical outcomes.

## Materials and methods

### Patients studied and diagnosis of PA

In this retrospective cross-sectional study, we consecutively included 123 patients with PA as a cohort used for modeling, who were referred to Kyushu University Hospital or Sapporo City General Hospital, underwent unilateral adrenalectomy, and had a postoperative follow-up of at least 6 months between January 2007 and November 2020. The exclusion criteria were patients with PA who failed to achieve complete biochemical success after adrenalectomy, assumed to be mainly due to misinterpretation of lateralization or incomplete resection of aldosterone-producing tissues, and patients with a postoperative follow-up shorter than 6 months. This study was part of the Kyushu Adrenal Network Database for Advanced medicine (Q-AND-A) study^18^. All patients were diagnosed with PA according to the guidelines of the Japan Endocrine Society^19^ and the Japanese Society of Hypertension^20^, with case detection, confirmatory tests, and subtype classification. At least one diagnostic confirmation test (saline infusion test or captopril challenge test) was performed based on the methods and criteria described in the above guidelines. More than two weeks prior to the diagnosis, antihypertensive drugs were routinely changed to calcium channel blockers and/or α-adrenergic blockers. Oral potassium supplementation was generally administered to patients with hypokalemia. For subtype diagnosis, adrenal venous sampling (AVS) with adrenocorticotropic hormone stimulation was performed and evaluated as described previously^21^. Unilateral adrenalectomy was performed upon request in patients with unilateral subtype of PA as determined by AVS and in those with clinically determined unilateral subtype of PA if AVS failed or AVS results were difficult to interpret. This retrospective study was approved by the institutional review board of Kyushu University (approval number: 2021–395) and was performed in accordance with relevant guidelines. Informed consent was obtained from the patients upon admission to our hospitals.

### Definition of biochemical and clinical outcomes

Biochemical and clinical outcomes were classified as complete, partial, or absent success and were defined according to the PASO study^5^. Briefly, patients with complete clinical success comprise those with normalized blood pressure levels without the use of antihypertensive medication after unilateral adrenalectomy. Patients with normalization of hypokalemia (if present preoperatively) and normalization of aldosterone-to-renin ratio were classified as having complete biochemical success.

### Model development by machine learning

Predictions were generated using a gradient-boosting machine model built with decision-tree base learners^22^. The advantages of using a decision tree are its ability to act strongly against outliers in the data and handle discrete features and missing values. We used the gradient-boosting predictor trained with the LightGBM^23^. Compared with other gradient-boosting predictors, LightGBM has better performance in terms of computational speed, memory consumption, and communication costs for parallel learning. Hyperparameters of the LightGBM were optimized using a grid search; the optimized values included max depth, learning rate, min data in leaf, num leaves, and n estimators. We selected clinical and biochemical data that are readily available in routine clinical practice as features among previously reported predictors^6–12^, including age, sex, BMI, duration of hypertension, defined daily dose (DDD) of antihypertensive medication, medical history of diabetes and PAC. We used a holdout method and randomly assigned patients with PA to two groups for analysis: training (80%) and test datasets (20%). Predictive models trained using stratified five-fold cross-validation on the training dataset were applied to the test dataset and their performance was evaluated. To evaluate predictive models, accuracy, positive predictive value, negative predictive value, and areas under the receiver operating characteristic curve (AUC) were calculated. Recently, in addition to the statistical validity of machine learning predictive models, their interpretability has been emphasized in the usefulness of the models^24^. We applied a Shapley additive explanations (SHAP) algorithm to our predictive model to obtain interpretations of the features that drive patient-specific predictions to mitigate the issue of black-box predictions^14,25^. SHAP is one of the well-established explainability methods for machine learning models, which are often difficult to interpret. SHAP is a model-agnostic representation of feature importance where the impact of each feature on a particular prediction is represented using Shapley values inspired by cooperative game theory. A Shapley value states, given the current set of feature values, how much a single feature in the context of its interaction with other features contributes to the difference between the actual prediction and the mean prediction. That is, the sum of the Shapley values for all features plus the mean prediction equals the actual prediction^26^. All the processes were implemented in Python using packages scikit-learn version 0.24.2^27^ and SHAP version 0.37.0^14^ libraries.

### Model validation in an external PA cohort

Model generalizability was assessed in an independent sample of patients with PA. An external cohort from National Hospital Organization Kyoto Medical Center, a single referral center in Japan, was used for validation. We identified 58 patients with PA who underwent unilateral adrenalectomy and had a postoperative follow-up between January 2007 to March 2019.

### Assay methods

The PAC used in the development of the model was measured using a commercial radioimmunoassay kit (SPAC-S Aldosterone; Fujirebio, Tokyo, Japan). The reference range of PAC for this kit in the supine position was 3.0–15.9 ng/dL. All other biochemical features were assayed in plasma or serum using standard methods.

### Statistical analysis

Clinical characteristics are presented as medians with interquartile ranges or counts with frequencies. Data between two groups were compared using the Mann–Whitney U test and Fisher’s exact test. Statistical analysis was performed using EZR statistical software (Saitama Medical Center, Jichi Medical University, Saitama, Japan), which is a graphical user interface for R (The R Foundation for Statistical Computing, Vienna, Austria, version 3.5.2)^28^. Statistical significance was set at *P* < 0.05.

## Results

### Clinical characteristics of the patients studied

**Figure 1:**
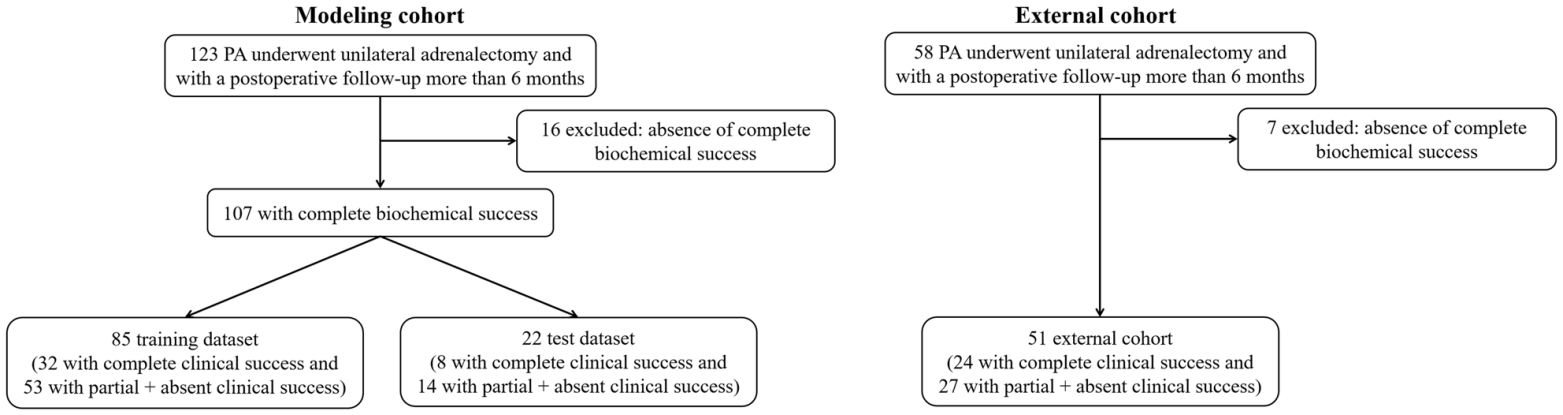
Flow chart.

A total of 107 patients with PA who achieved complete biochemical success after adrenalectomy were included for modeling in this study (Fig. 1). Based on the PASO study, of the 107 patients studied, 40, 38, and 29 patients achieved complete clinical success, partial clinical success, and absent clinical success, respectively. The clinical and biochemical characteristics of the patients in the modeling cohort are presented in Table 1. Patients who achieved complete clinical success were younger, more frequently female, had lower BMI, shorter duration of hypertension, and lower DDD of antihypertensive drugs than those who achieved partial and absent clinical success. In the following analysis, 85 (32 complete clinical success and 53 partial plus absent clinical success) and 22 (8 complete clinical success and 14 partial plus absent clinical success) patients were randomly assigned to the training and test datasets, respectively. We included 51 out of 58 patients with PA in the external cohort and excluded 7 patients who failed to achieve complete biochemical success after adrenalectomy from further analysis (Fig. 1). Clinical and biochemical characteristics of the modeling and external cohorts are shown in Table 2.

**Table 1:** Preoperative clinical characteristics of 107 patients with primary aldosteronism in the modeling cohort.

Variables	Complete clinical success (n = 40)	Partial + absent clinical success (n = 67)	*P* value
Age at diagnosis (years)	50 (42–59)	56 (48–63)	0.029
Sex (female; %)	25 (62.5)	25 (37.3)	0.016
BMI (kg/m^2^)	22.3 (20.4–24.9)	24.7 (22.8–27.0)	0.005
Systolic blood pressure (mmHg)	136 (124–142)	140 (130–154)	0.067
Diastolic blood pressure (mmHg)	80 (71–90)	82 (75–92)	0.307
Duration of hypertension (years)	5 (1–10)	10 (6–20)	< 0.001
Antihypertensive medication (DDD)	1.0 (0.7–2.0)	2.3 (1.3–3.5)	< 0.001
PAC (ng/dL)	38.6 (22.2–50.3)	31.3 (19.8–37.2)	0.219
Plasma renin activity (ng/mL/h)	0.20 (0.10–0.30)	0.20 (0.10–0.30)	0.509
Lowest serum potassium (mEq/L)	2.9 (2.4–3.3)	2.9 (2.6–3.2)	0.789
Medical history of diabetes (Yes; %)	5 (12.5)	11 (16.4)	0.780

Data are expressed as medians with interquartile ranges or numbers with percentages. BMI, body mass index; DDD, defined daily dose; PAC, plasma aldosterone concentration.

**Table 2:** Patient clinical characteristics of the modeling and external primary aldosteronism cohort.

Variables	Modeling cohort (n = 107)	External cohort (n = 51)	*P* value
Clinical success (complete; %)	40 (37.4)	24 (47.1)	0.299
Age at diagnosis (years)	54 (45–62)	46 (40–58)	0.035
Sex (female; %)	50 (46.7)	24 (47.1)	1.000
BMI (kg/m^2^)	24.2 (21.8–26.4)	23.3 (20.9–27.1)	0.449
Systolic blood pressure (mmHg)	138 (128–151)	133 (125–146)	0.180
Diastolic blood pressure (mmHg)	81 (73–92)	80 (75–92)	0.997
Duration of hypertension (years)	10 (4–15)	6 (2–10)	0.015
Antihypertensive medication (DDD)	2.0 (1.0–2.8)	2.3 (1.5–3.2)	0.043
PAC (ng/dL)	32.4 (20.8–48.6)	34.2 (21.7–51.3)	0.666
Plasma renin activity (ng/mL/h)	0.20 (0.10–0.30)	0.30 (0.20–0.50)	< 0.001

Data are expressed as medians with interquartile ranges or number with percentage. BMI, body mass index; DDD, defined daily dose; PAC, plasma aldosterone concentration.

### Machine learning-based model for predicting complete clinical success

**Figure 2:**
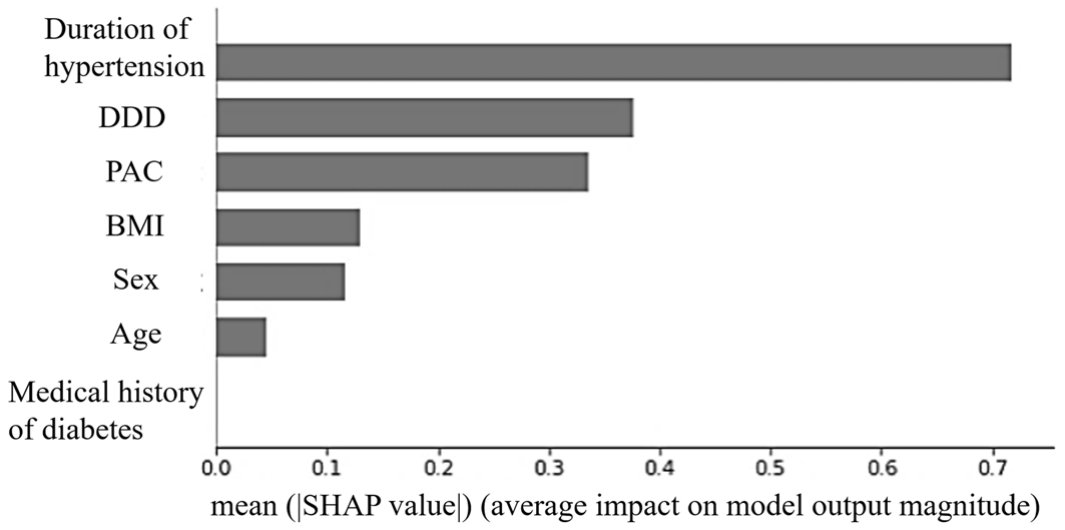
Mean absolute SHAP value of seven clinical features in the machine learning-based predictive model for complete clinical success.

**Figure 3:**
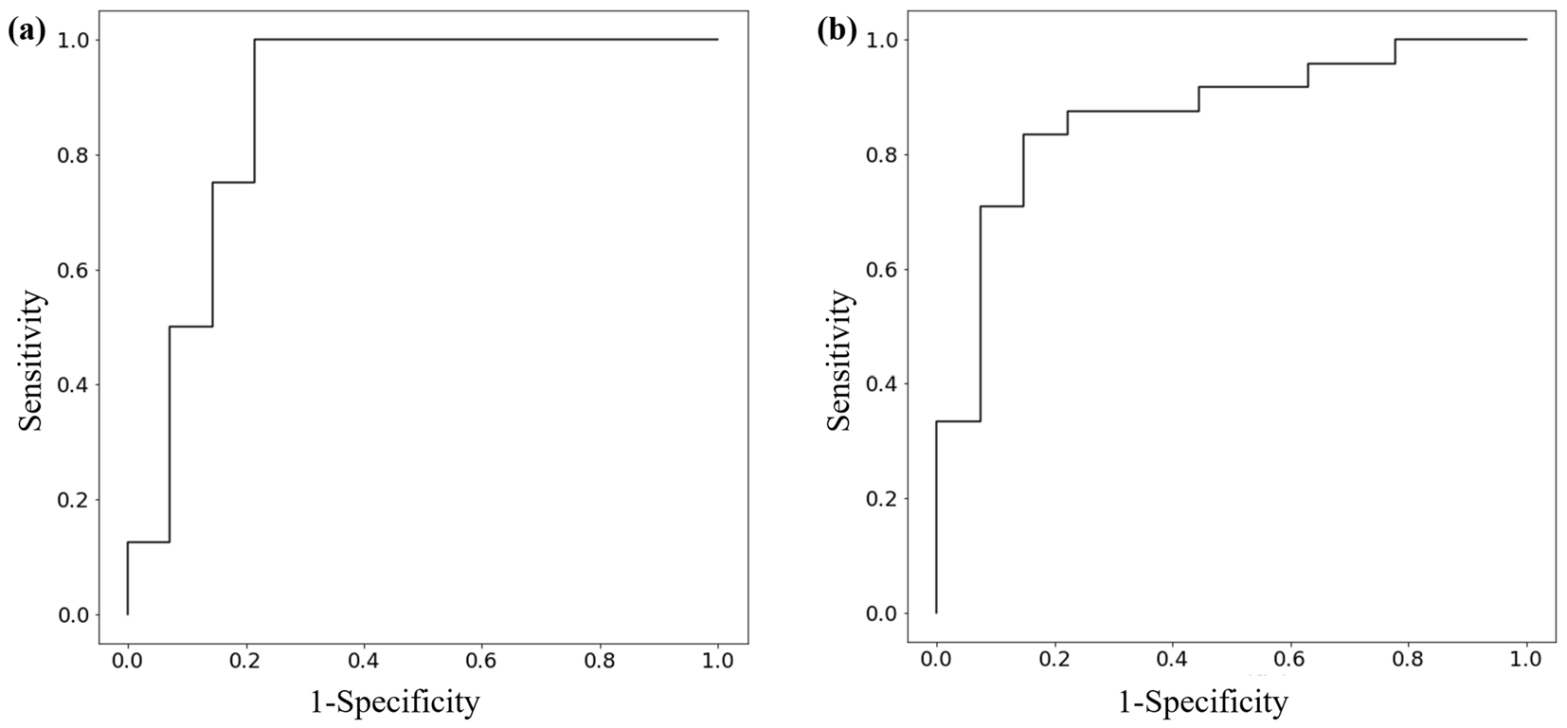
Receiver operating characteristic curves for the model predicting complete clinical success in **(a)** the test dataset (n = 22) and **(b)** the external cohort (n = 51).

We first developed a machine learning-based model using seven clinical features. Figure 2 shows the mean absolute SHAP value of each feature in the model. In this study, a higher SHAP value for a feature was interpreted as a higher probability of complete clinical success after unilateral adrenalectomy. The rank order of the mean absolute SHAP values across the seven features in the developed model reflects their importance. For the prediction of complete clinical success, the rank order was the duration of hypertension, followed by DDD of antihypertensive medication and PAC, whereas the medical history of diabetes did not contribute at all to the prediction in this model (mean absolute SHAP value = 0). To improve the predictive performance, reduce the effect of the curse of dimensionality, and shorten learning time^29^, we removed the medical history of diabetes from the list of features and redeveloped the model. The predictive accuracy, positive predictive value, negative predictive value, and AUC for the model developed using the six features in the test dataset were 77.3%, 80.0%, 76.5%, and 0.884 (95% confidence interval: 0.737–1.000), respectively. Figure 3a shows the receiver operating characteristic curve of the model.

### Validation of the predictive model in the external cohort

Our predictive model with the six features was validated using the independent external cohort. As a result, the predictive accuracy, positive predictive value, negative predictive value, and AUC were 80.4%, 81.8%, 79.3%, and 0.867 (95% confidence interval: 0.763–0.971), respectively. Figure 3b shows the receiver operating characteristic curve of the model. Furthermore, this performance was roughly comparable to that obtained with the test dataset, suggesting that the effect of overfitting in our model was minimal.

### Interpretation and evaluation of the machine learning-based model

**Figure 4:**
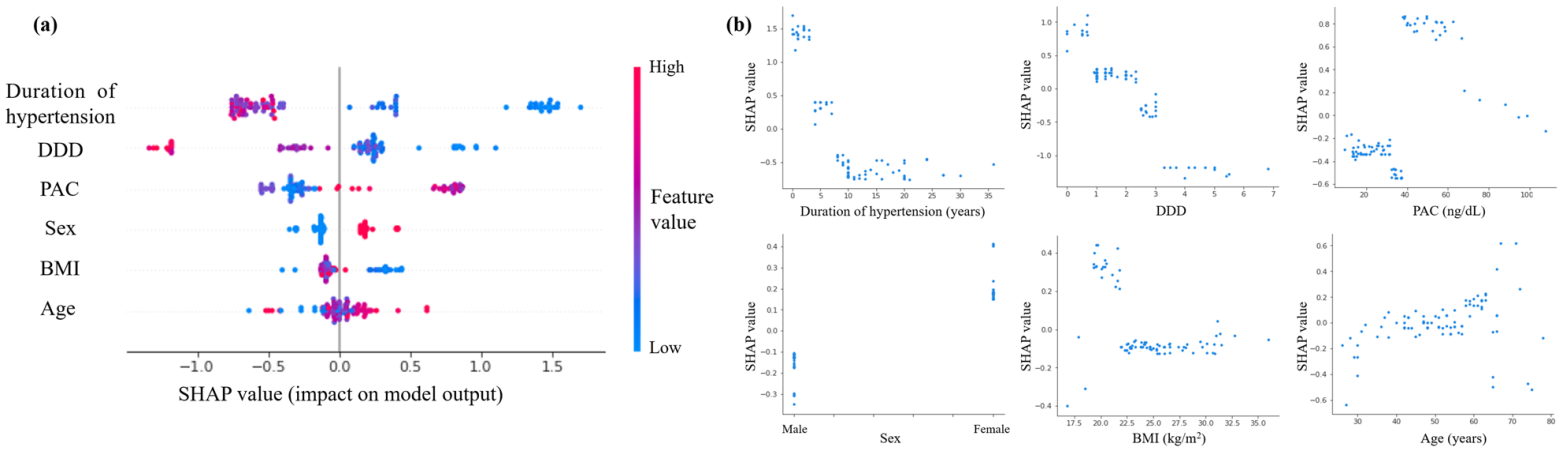
SHAP summary plot and dependence plots. **(a)** SHAP summary plot of six features in the model. Features were sorted in descending order by SHAP values. SHAP values for each feature were calculated for each patient-derived model, which is represented by a single dot. Dots were colored based on the underlying feature’s value. For the features of sex, the red dots indicated female and the blue dots indicated male. **(b)** SHAP dependence plots for each feature in the model. SHAP values for specific features exceed zero, representing an increased probability of complete clinical success.

The SHAP summary plot and SHAP dependence plots of the model developed using the six features were used for interpreting the relationship between the values of each feature and predicting complete clinical success (Fig. 4). As shown in Fig. 4a, predicting complete clinical success was found to be associated with a shorter duration of hypertension, lower DDD of antihypertensive medication, higher PAC, female sex, and lower BMI. However, there was no consistent trend in age. The duration of hypertension, DDD of antihypertensive medication, and BMI were negatively and non-linearly related to the prediction of complete clinical success (Fig. 4b). An almost linear negative correlation was observed when the duration of hypertension was less than 7 years, and a constant low probability of complete clinical success was observed when the duration was more than 7 years. Similarly, an almost linear negative correlation was observed when DDD of antihypertensive medication was less than 3, and a constant low probability of complete clinical success was observed when DDD was more than 3. PAC contributed to the prediction of complete clinical success at 37 ng/dL and above, whereas the effect was smaller at 67 ng/dL and above. BMI contributed to the prediction of complete clinical success at 22 kg/m^2^ or less, whereas it showed a constant low probability of 22 kg/m^2^ or more.

**Figure 5:**
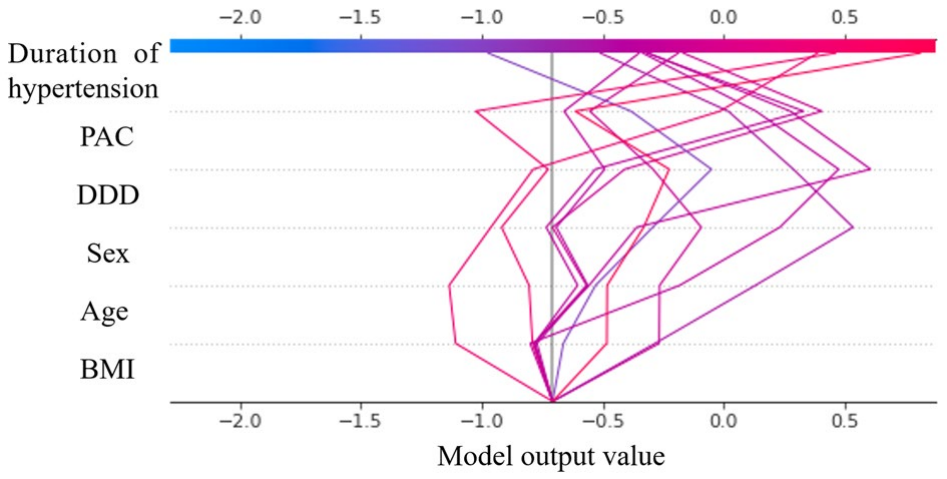
SHAP decision plot of patients misclassified in the developed model. The vertical axis of the decision plot showed each feature of the predictive model. The colored prediction lines started at the bottom of the plot and show how the SHAP values accumulate from the base value to arrive at the model’s final score at the top of the plot. The prediction lines tended to make drastic turns at feature with high importance and reached the estimated probability of complete clinical success.

SHAP decision plots offer a detailed view of a model’s inner workings and show how models make decisions. In Fig. 5, we synthesized prediction lines for patients in the training dataset who were misclassified by our predictive model. When the more influential feature, hypertension duration, the individual prediction lines changed significantly, leading to misclassification of the final risk score. Of the six features used in the developed model, the duration of hypertension is often difficult to estimate accurately and may have misestimated in the prediction of complete clinical success for some patients.

## Discussion

In this study, we developed a machine learning-based model for predicting complete clinical success in patients with PA using preoperative clinical data readily available in routine clinical practice. Our predictive model was about 80% accurate on both the test dataset and the external cohort. Altogether, these observations suggest that our model has a high predictive value for complete clinical success after adrenalectomy. We found six features of high predictive importance in the model and clarified the relationship between these features and clinical outcomes. Because more than half of patients with PA who undergo surgical treatment do not achieve complete clinical success, our model can provide useful information for clinicians and patients with PA in making treatment decisions. This model should also help clinicians identify patients who need close follow-up, because patients who do not achieve complete clinical success after unilateral adrenalectomy may have residual hypertension and require continuous monitoring after surgery.

Although unilateral subtype of PA can be cured by unilateral adrenalectomy, biochemical and clinical success are not often coincide^5^, which may discourage patients with unilateral subtype of PA from undergoing adrenalectomy even when appropriate. Indeed, of those with unilateral subtype of PA determined by AVS, 21.8% in Japanese centers and 8.6% in European centers reportedly did not undergo adrenalectomy^30^. Thus, the application of this model to patients with unilateral subtype of PA can help correlate their clinical data effectively with clinical outcomes, thereby leading to a reduction in the number of those who are reluctant to undergo adrenalectomy.

Several predictors related to postoperative clinical outcomes in PA have been reported^6–12^. However, the reported predictors were not often consistent across studies. This may be partly because previous studies assumed the linearity of the features and used generalized linear models. In this study, we comprehensively incorporated clinical data that are readily available in routine clinical practice among predictors reported to be related to clinical outcomes in the non-linear machine learning algorithm. As a decision tree-based model, LightGBM also has the advantage of being robust against multicollinearity^31^. When features were selected to improve the performance of the developed predictive model, six important features were found in the following order: duration of hypertension, DDD of antihypertensive medication, PAC, sex, BMI, and age. Due to the different test datasets used in each study, it is difficult to make a direct comparison of the accuracy between our developed predictive model and previously reported scoring models. However, in the test dataset of each study, the predictive accuracy, positive predictive value, and negative predictive value of our predictive model were 77.3%, 80.0%, and 76.5%, respectively, compared to 65.7%, 75.0%, and 63.6%, respectively, for the aldosteronoma resolution score (ARS) (≥ 4 points)^12^ and 73.0%, 64.4%, and 80.0%, respectively, for the primary aldosteronism surgical outcome (PASO) score (> 16 points)^7^. The performance of our predictive model compared favorably with those reported previously. We could confirm the usefulness of applying a non-linear machine learning algorithm in predicting clinical outcomes in PA.

**Figure 6:**
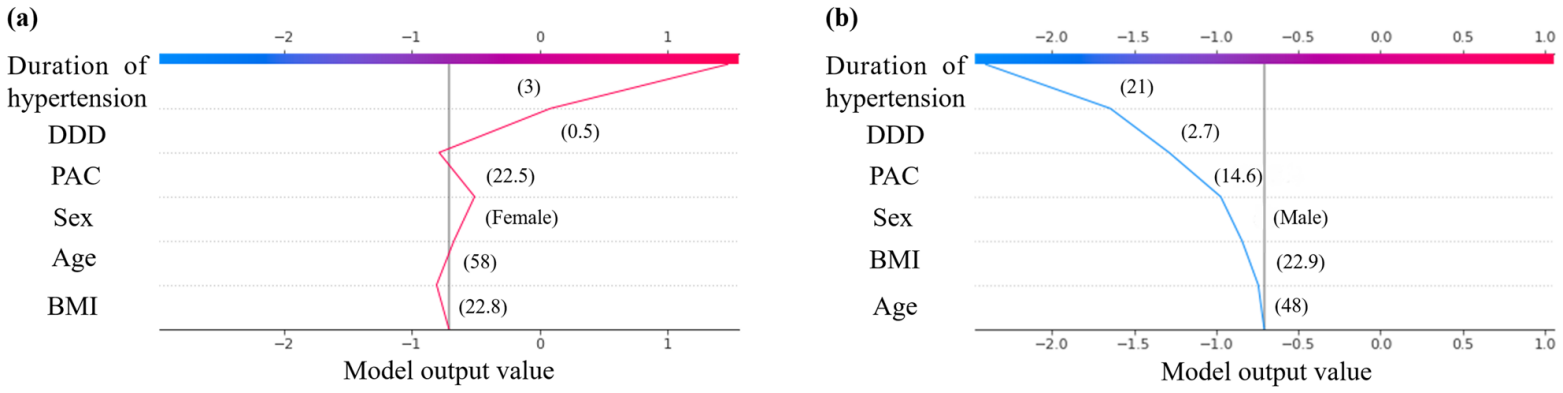
SHAP decision plots for two cases **(a, b)** in the dataset. The prediction line represented the pathway leading to the estimated probability of complete clinical success for each patient in our predictive model.

The advantage of our study is the use of machine learning to reveal the non-linear relationship between clinical features and complete clinical success, and the use of SHAP values to uncover the black box of the machine learning-based model. Visualizing the change in SHAP values can help understand how these features affect the output of the predictive model (Fig. 4). In this study, the duration of hypertension, DDD of antihypertensive medication, PAC, and BMI showed a non-linear relationship with complete clinical success. Our predictive model can provide the importance of the features involved in the prediction by showing as a SHAP decision plot how the model uses the features to make decisions for each patient used. This visual description allows the clinician to immediately and easily interpret the output of the model for each patient. For example, Fig. 6 shows the SHAP decision plots for two cases in the dataset. In case (a), although the SHAP values for PAC and BMI were negative, the prediction of complete clinical success was derived with a particularly large effect of duration of hypertension and DDD. In case (b), the SHAP values of all six features were negative, predicting that complete clinical success would not be achieved. As predicted by the model, postoperatively, blood pressure levels normalized in case (a), while hypertension remained in case (b). The duration of hypertension was the most relevant feature to complete clinical success. In addition, the persistence of hypertension due to excessive aldosterone secretion over a long-time period could have detrimental and irreversible effects on the cardiovascular system. The mechanistic relationship between the duration of hypertension and clinical outcomes must await further investigation.

There are several limitations to this study. First, because this study retrospectively investigated patients with PA who underwent unilateral adrenalectomy, prospective studies are required to assess the validity of our predictive model. Second, the model developed cannot distinguish between patients with partial clinical success and those with absent clinical success, although they both require postoperative antihypertensive medication and continuous blood pressure control. Third, patients with PA who failed to achieve complete biochemical success were excluded from this study, and the performance of the predictive model when they are included is unknown and is a subject of future research. Finally, the model was developed based on Japanese clinical practice guidelines and was not validated for populations of other races. The influence of different diagnostic methods and subtype testing of PA may result in bias.

In conclusion, we developed a machine learning-based model to predict complete clinical success after unilateral adrenalectomy. Moreover, our predictive model is based on readily available clinical features, which may be used with ease in routine clinical practice. The developed predictive model may be useful in assessing the benefit of unilateral adrenalectomy and in selecting surgical treatment and antihypertensive medication for patients with PA in clinical practice.

## Data Availability

The datasets generated and analyzed during the current study is not publicly available but are available from the corresponding author on reasonable request. The processed codes are available from GitHub: https://github.com/hiroki-kane/Clinical-Outcomes-of-PA.
